# The heart of celiac disease: understanding dilated cardiomyopathy, pathophysiology, and care—a systematic review

**DOI:** 10.1186/s43044-024-00534-x

**Published:** 2024-08-16

**Authors:** Rajesh Yadavalli, Sarosh Nawaz, Abdulaziz Mohammed Althwanay, Esraa M. AlEdani, Harleen Kaur, Malik Kasapoglu, Pousette Farouk Hamid

**Affiliations:** 1 Rajiv Gandhi Institute of Medical Sciences, Adilabad, 504001 India; 2 Pakistan Institute of Medical Sciences, Islamabad, Pakistan; 3 Imam Abdulrahman Bin Faisal University, Dammam, Saudi Arabia; 4 University of Basra, Basra, Iraq; 5 Maharishi Markandeshwar Institute of Medical Sciences and Research, Mullana, India; 6 Bahcesehir University School of Medicine, Istanbul, Turkey; 7 Ain Shams University, Cairo, Egypt; 8 Department of Internal Medicine, California Institute of Behavioral Neurosciences and Psychology, Fairfield, USA

**Keywords:** Celiac disease, Dilated cardiomyopathy, Cardiac manifestations, Gluten-free diet, Management, Systematic review

## Abstract

**Background:**

Cardiac manifestations are infrequently reported in association with celiac disease, but clear link has not been established. The aim of this study was to explore the connection of dilated cardiomyopathy in celiac disease. This systematic review also provides a comprehensive overview of the association between celiac disease and various cardiac manifestations with pathophysiology and management.

**Main body:**

We searched PubMed, Cochrane Library, Google Scholar, Embase, Scopus, and Springer nature databases through June 4th 2023 for preferred studies related to our topic using MeSH and Regular keywords. After comprehensive search analysis, data extraction and quality appraisal 19 studies were included in the study. Although results varied across studies, majority of the studies revealed a positive link. Notably, some studies suggested an association between celiac disease and dilated cardiomyopathy, while others did not. These discrepancies could be attributed to differences in methodologies, study populations, and regional variations. Several studies have shown the association of various cardiac manifestations in celiac disease.

**Conclusion:**

Although dilated cardiomyopathy is associated with celiac disease in majority of the studies, there are also studies that conflict with the association. The complex relationship between celiac disease and cardiovascular manifestations potentiates the need for further research with standardized methodologies, larger sample sizes, and consideration of regional variations. Such insights are vital for improving clinical practice by establishing preventive strategies, active screening, early diagnosis, mitigating risks which helps in optimizing cardiovascular health in individuals with celiac disease.

**Supplementary Information:**

The online version contains supplementary material available at 10.1186/s43044-024-00534-x.

## Background

Celiac disease (CD), also known as gluten-sensitive enteropathy, is a chronic multisystem autoimmune disease. It mainly affects the small intestine in genetically predisposed individuals due to their sensitivity to dietary gluten and related proteins. These gluten proteins are major components of cereals—wheat, barley, and rye [1–3]. The estimated global prevalence of CD is approximately 0.5 to 1% in the general population. Individuals with autoimmune disorders or first-degree relatives have a significantly higher risk of developing CD [4, 5]. Nearly 1 in 133 Americans are affected by CD [6]. CD can affect individuals of all races and ages but is approximately twice as common in females than in males. This gender difference may be attributed to the higher prevalence of autoimmune diseases in females [7]. HLA-DQ2 and HLA-DQ8 are strongly associated with genetic susceptibility to CD [8].

CD is characterized by mucosal inflammation, crypt hyperplasia, and villous atrophy, resulting in malabsorption symptoms [2]. Only 27% of adults with CD exhibit classical symptoms [9]. CD is termed as a “celiac iceberg” with varied disease presentation from malabsorption symptoms in classical form to asymptomatic silent form. It takes nearly 6–10 years to get diagnosed with CD [6]. It is believed that for every diagnosed patient there are more than five undiagnosed [10]. Asymptomatic CD occurs when patients do not exhibit symptoms but still experience villous atrophy damage to their small intestine [5, 10]. CD is associated with a wide range of extra-intestinal manifestations [11]. 2% of patients with CD were shown to present with non-classical symptoms [8]. 83% of estimated Americans with CD are undiagnosed or misdiagnosed [6]. CD is associated with an overall 1.2 to twofold increase in mortality mainly caused by lymphoproliferative and gastrointestinal malignancies [12].

Cardiac manifestations are increasingly reported associated with CD which includes dilated cardiomyopathy (DCM), myocarditis, arrhythmias, pericardial effusion, myocardial infarction, and heart failure. DCM is infrequently reported to be associated with CD, but a clear association has not been met [13]. DCM is a disease of the heart that mainly affects the left ventricle and is seen as left ventricular dilation that is concerned with systolic dysfunction in the absence of other etiologies like hypertension, coronary disease, or valvular dysfunction [14]. The pathogenesis of DCM in CD is unclear. Several mechanisms have been proposed that likely explain the etiology and progression of DCM. These include nutritional deficiencies leading to cardiomyopathy due to chronic malabsorption and direct myocardial injury from immune response to antigens present in both the myocardium and small intestine [13]. Understanding the theories by which cardiomyopathy arises helps to identify the cause and prevent the occurrence in future patients [13]. Long-term strict adherence to a gluten-free diet has shown marked improvement in the course of disease [15].

This systematic review aims to find the association of dilated cardiomyopathy with celiac disease and cardiac manifestations and complications in celiac disease. The co-occurrence of CD and dilated cardiomyopathy can complicate the clinical management of these conditions. Understanding the potential connection between CD and Dilated cardiomyopathy is of paramount importance for clinicians and healthcare providers. Identifying CD in patients with DCM may enable timely treatment and dietary modifications, potentially improving cardiac health. Conversely, recognizing DCM in individuals with CD can lead to the early detection of heart complications and appropriate interventions. If our review establishes a significant association between these conditions, it could lead to the development of targeted screening protocols and preventive strategies. These strategies may prove invaluable in identifying at-risk individuals and preventing or mitigating the progression of cardiac complications. Clinicians can provide more comprehensive care by considering potential cardiac complications in CD patients, leading to better overall health management and reducing mortality risk. Additionally, this study intends to review the pathogenesis and management of DCM in CD.

## Methods

The systematic review was conducted as per guidelines of The Preferred Reporting Items for Systematic Review and Meta-Analyses (PRISMA 2020).

### Study protocol

We performed preliminary searches on our research question. We then prepared our protocol according to the guidelines of preferred reporting items for systematic review and meta-analyses protocol (PRISMA 2020).

### Data sources and search strategy

Two investigators (RY and SN) personally reviewed the databases. We reviewed PubMed, Cochrane Library, Google Scholar, Embase, Scopus, and Springer nature databases through June 4th 2023 for preferred studies related to our topic using MeSH and Regular keywords. We customized our search to include any clinical studies related to our study with keywords, inclusion, and exclusion criteria.

Medical subject headings (MeSH) keywords used in PubMed search were celiac disease OR gut OR tropical sprue OR gut sensitive enteropathy OR gluten free diet AND dilated cardiomyopathy OR heart OR cardiac condition OR arrhythmia OR myocarditis AND (("Celiac Disease/complications"[Majr] OR "Celiac Disease/pathology"[Majr])) OR ("Celiac Disease/complications"[Majr:NoExp] OR "Celiac Disease/pathology"[Majr:NoExp]) AND (("Cardiomyopathy, Dilated/complications"[Majr] OR "Cardiomyopathy, Dilated/etiology"[Majr] OR "Cardiomyopathy, Dilated/pathology"[Majr])) OR ("Cardiomyopathy, Dilated/complications"[Majr:NoExp] OR "Cardiomyopathy, Dilated/etiology"[Majr:NoExp] OR "Cardiomyopathy, Dilated/pathology"[Majr:NoExp]).

Keywords used in Embase, Google scholar, Scopus. Cochrane Library, Springer nature: “celiac disease” and “dilated cardiomyopathy.”

*Inclusion criteria*: Free full-text articles published in and after 2013 and written in English language related to the question were included.

*Exclusion criteria*: unpublished literature, gray literature, abstracts only papers, or non-English language papers.

### Study screening, extraction, and selection

After the search strategy was completed, all articles were downloaded in excel or csv file and imported into Endnote software. Then duplicates were removed, and studies are screened independently by two separate reviewers using a standardized form. Extracted data included study characteristics (author, year, country, study design), participant details (sample size, age, gender), and key findings (outcomes, effect estimates, confidence intervals). The extracted data were cross-checked for accuracy, and any disagreements were resolved through discussion.

### Quality appraisal

Various quality assessment tools were based on the type of study (Table 1). The Joanna Briggs Institute checklist was used for quality appraisal of case report, while that Scale for the Assessment of Narrative Review Articles-SANRA checklist was used for narrative review articles and letters to the editor. AMSTAR checklist was used for assessing quality of systematic reviews. The Newcastle–Ottawa Scale was used to assess the quality of observational studies. For all the studies satisfying the criteria of >  = 70% were only included in the study (Table 1). The overall validity and reliability of the conclusions drawn from systematic reviews depend heavily on the quality of included studies which are quantified through quality appraisal score not much variability in the interpreted scores and studies were found.

**Table 1:** Quality assessment of the included studies

First author	Publication year	Report type	Quality assessment tool used	Overall score (Included or not)
Hidalgo et al. [16]	2020	Systematic review and meta-analysis	AMSTAR	9/11 (Included)
Schmucker et al. [38]	2022	Systematic review and meta-analysis	AMSTAR	9/11 (Included)
Rashidinia et al. [24]	2021	Observational study	Newcastle Ottawa scale	8/8 (Included)
Karadas et al. [19]	2016	Observational study	Newcastle Ottawa scale	8/8 (Included)
Ibrahim et al. [13]	2023	Observational study	Newcastle Ottawa scale	8/8 (Included)
Lebwohl et al. [18]	2015	Observational study	Newcastle Ottawa scale	8/8 (Included)
Noori et al. [21]	2016	Case control study	Newcastle–Ottawa scale	6/8 (Included)
Huang et al. [20]	2022	Case control study	Newcastle Ottawa scale	8/8 (Included)
Wang et al. [17]	2023	Narrative review	SANRA checklist	6/6 (Included)
Mehra et al. [26]	2022	Case reports	JBI checklist	7/8 (Included)
Bohra et al. [25]	2020	Case reports	JBI checklist	7/8 (Included)
Myrmel et al. [23]	2021	Case reports	JBI checklist	7/8 (Included)
Samy et al. [27]	2017	Case reports	JBI checklist	7/8 (Included)
Ashrafi et al. [35]	2014	Case reports	JBI checklist	7/8 (Included)
Saada et al. [29]	2017	Case reports	JBI checklist	7/8 (Included)
Elnour et al. [12]	2017	Case reports	JBI checklist	7/8 (Included)
Patel et al. [37]	2018	Case reports	JBI checklist	7/8 (Included)
McGrath et al. [34]	2016	Case reports	JBI checklist	7/8 (Included)
Mannarino et al. [28]	2022	Case reports	JBI checklist	7/8 (Included)

Newcastle Ottawa scale accepted score (> = 70%): minimum score 6 out of 9; AMSTAR checklist accepted score (> = 70%): minimum score 8 out of 11; SANRA Checklist accepted score (> = 70%): minimum score 5 out of 6; JBI Checklist accepted score (> = 70%): minimum score 6 out of 8;

AMSTAR: A Measurement Tool to Assess systematic Reviews, SANSA: Scale for the Assessment of Narrative Review Articles, JBI: Joanna Briggs Institute

### Analysis of outcome

The primary outcome of interest was the association between CD and DCM. The secondary outcome was the association between CD and cardiac symptoms, pathophysiology, and management.

## Results

### Search result

**Fig. 1:**
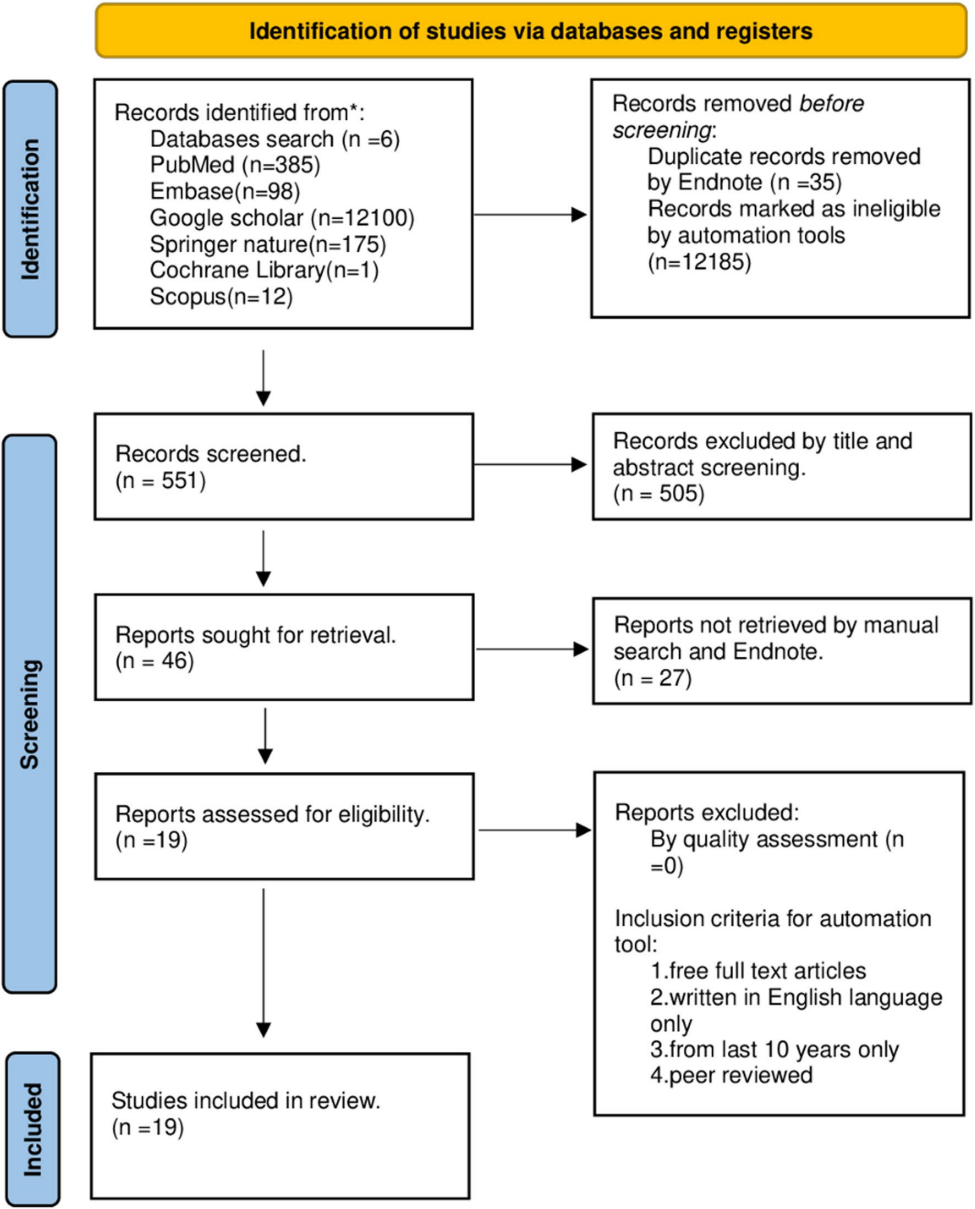
Overview of the study review process according to the PRISMA (2020) flow diagram

Initial screening of following databases yielded in 12,771 records (Fig. 1). Out of which 35 duplicates were removed, 12,185 records were excluded through automation tools based on inclusion and exclusion criteria. Of the 551 records that are relevant to study, 505 records were excluded based on screening through title and abstract. Out of 46 reports that are sought for retrieval, 27 were not retrieved based on manual search and Endnote. The remaining 19 articles were included in the assessment of quality, and all 19 articles were finally included in the study following quality assessment and eligibility criteria (Fig. 1).

### Study characteristics

A total of 19 studies were included in the review, which included 2 systematic review and meta-analyses, 1 literature reviews, 2 case–control studies, 4 cross-sectional studies, and 10 case reports (Table 2).

**Table 2:** Study characteristics of the included studies/reports

First author	country	study type	purpose of study	number of patients/studies	Population	Celiac disease diagnosis	Main findings
Noori et al. [21]	Iran	Case–control	to evaluate the relationship between CD and dilated cardiomyopathy in children	38	Children (1–18 years)	Serology only	Screening for CD is strongly recommended in children with dilated cardiomyopathy
Rashidinia et al. [24]	Iran	Cross-sectional	to evaluate the relationship between CD and dilated cardiomyopathy in children through serological screening in correlation with ejection fraction	123	Adults (18 to 80 years)	Serology only	Screening for CD is strongly recommended in children with dilated cardiomyopathy
Wang et al. [17]	USA	literature review	to investigate the association between the CD and cardiovascular disease	12 Studies	Mixed	Biopsy/serology	It suggests that individuals with CD may have a higher risk of cardiovascular disease compared with the general population
Huang et al. [20]	China	Case–control study	To find the casual association between CD and cardiovascular problems using Mendelian randomization approach among European ancestry	12,041	Mixed	Biopsy/serology	CD itself has no causal effect on cardiovascular manifestations among European ancestry
Karadas et al. [19]	Turkey	cross-sectional cohort study	to investigate the effects of CD on cardiac function in children and effect of gluten-free diet on heart	26	Children (1–18 years)	Biopsy/serology	early determination of the cardiac function helps in monitoring and follow-up which may help to show the effectiveness of treatment
Ibrahim et al. [13]	Egypt	Cross-sectional study	to evaluate the myocardial dysfunction in patients with CD	42	Children (6 months –18 years)	Biopsy/serology	Children with CD had subclinical myocardial dysfunction, especially in RV which is better detected by tissue Doppler imaging. These dysfunctions are increased with the presence of extra-intestinal manifestations
Hidalgo et al. [16]	USA	Systematic review and meta-analysis	To find the association between CD and atrial fibrillation	4 studies	Mixed	Biopsy/ serology	38% increased risk of atrial fibrillation in patients with CD as compared to individuals without CD used as controls
Lebwohl et al. [18]	USA	A population-based study	To find histological evidence of CD from 28 pathology centers in Sweden	7440	Mixed	Biopsy	persistent Villous atrophy on follow-up biopsy was not associated with an increased risk of IHD or AF. Failed mucosal healing does not influence the risk of these cardiac events
Schmucker et al. [38]	Germany	Systematic review and meta-analysis	Is a gluten-reduced or gluten-free diet effective for the primary prevention of cardiovascular disease?	4 studies	Mixed	Biopsy/serology	significant decreased risk of cardiovascular morbidity with gluten-free diet
Mehra et al. [26]	India	Case report	Child with congestive heart failure in CD	–	10y/M	Serology and biopsy	Children with dilated cardiomyopathy or myocarditis with no known etiologies should be thoroughly investigated for CD
Bohra et al. [25]	India	Case report	CD-associated cardiomyopathy whose cardiac function improved substantially after treatment with a gluten-free diet	–	35y/F	Serology and biopsy	Cardiomyopathy associated with CD and celiac crisis is a serious and potentially lethal condition. However, with early diagnosis and treatment with a gluten-free diet, steroids cardiomyopathy in patients with CD may be completely reversible
Mannarino et al. [28]	Italy	Case report	Association between atrioventricular block and CD	–	4y/F	Serology and biopsy	clinical presentation of CD in pediatric age as extra-intestinal manifestations of the cardiac involvement should be considered
Myrmel et al. [23]	Norway	Case report	To evaluate myocarditis as an extra-intestinal manifestation of CD	–	28y/M	Serology and biopsy	patients with myocarditis and iron deficiency anemia or other signs of malabsorption should be screened for CD
Ashrafi et al. [35]	Iran	Case report	Association between asymptomatic pericardial effusion and CeD	–	40y/M	Serology and biopsy	Asymptomatic pericardial effusion may be seen in adults with CD
Samy et al. [27]	Canada	Case report	relation between CD and Cardiac conduction disease	–	42/F	Serology and biopsy	Association between CD and cardiac diseases such as dilated cardiomyopathy or unexplained arrhythmia
Saada et al. [29]	Tunisia	Case report	association of hypothyroidism and CD	–	23y/F	Serology and biopsy	The possibility of CD should be considered in patients with hypoparathyroidism and pericarditis that seems unduly difficult to treat
Elnour et al. [12]	United Arab Emirates	Case report	Extra-intestinal manifestations of CD as dilated cardiomyopathy	–	33y/F	Serology and biopsy	In patients with dilated cardiomyopathy and iron deficiency anemia, screening for CD is important
Patel et al. [37]	UK	Case report	Iron deficiency anemia with global ventricular involvement as presentation in CD	–	19y/M	Serology and biopsy	Adequate screening should be done in all patients with dilated cardiomyopathy; especially, index of suspicion should be raised in those with concomitant nutritional deficiencies
McGrath et al. [34]	UK	case report	Link between coeliac disease and idiopathic dilated cardiomyopathy	–	57y/M	Serology and biopsy	Testing for CD should be considered in unexplained dilated cardiomyopathy

Characteristics of included studies in the systematic review

## Discussion

### Pathophysiology

Several mechanisms have been proposed to possibly explain the pathophysiology of cardiac manifestations in CD. Firstly, the immune system is implicated as a major contributor, involving inflammation and oxidative stress [16]. Wang et al. similarly suggest an inflammatory pathway. They propose that a cumulative role of molecular mechanisms, inflammatory pathways, endothelial dysfunction, and genetic susceptibility factors is associated with cardiac manifestations in CD [17].

**Fig. 2:**
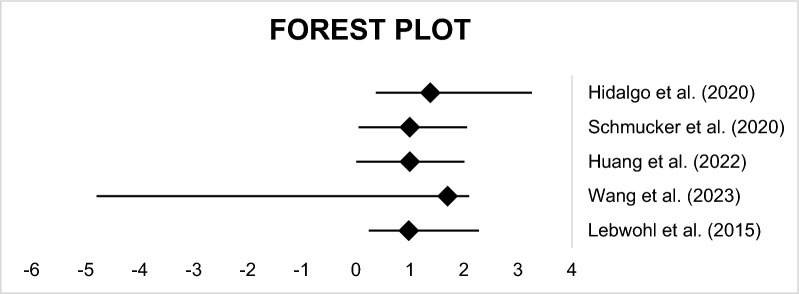
Forest plot: Forest plot for the hazard ratios/ odds ratios. A brief description of diagram including effect size, confidence interval, and statistical significance

Additionally, inflammation plays a crucial role in the pathogenesis of both CD and cardiac disease. In CD patients, the ingestion of gluten activates the immune system, leading to the production of pro-inflammatory cytokines such as tumor necrosis factor-alpha (TNF-α), interleukin-1 (IL-1), and IL-6. These cytokines contribute to the development of intestinal inflammation, villous atrophy, and crypt hyperplasia observed in CD [17]. Furthermore, these cytokines and activated immune cells may influence the contractility and electrical stability of myocytes, resulting in fibroblast activation and cellular fibrosis [16]. Lebwohl et al. and Karadas et al. provide additional support for the role of chronic inflammation in cardiac manifestations [18, 19] (Fig. 2).

Endothelial dysfunction serves as an early indicator of atherosclerosis, leading to vessel narrowing and development of a pro-inflammatory–prothrombotic state. Studies have shown that individuals with CD exhibit signs of endothelial dysfunction, including impaired flow-mediated dilation and elevated levels of endothelial activation markers such as von Willebrand factor and soluble intercellular adhesion molecule-1. These findings suggest that endothelial dysfunction in CD patients may influence the development of cardiac disease. Additionally, genetic risk factors, such as HLA (human leukocyte antigen) haplotypes, may establish a connection between cardiac conditions and CD [17]. However, Huang et al. suggest that there is no increased risk of coronary heart disease associated with genetic variants related to CD [20].

Furthermore, another significant mechanism involves autoimmunity, a process characterized by antigen mimicry that triggers an autoimmune response targeting self-antigens in various tissues, including the myocardium and small intestine [21–26]. Elnour et al. proposed a mechanism wherein severe nutritional deficiencies arise due to chronic malabsorption [12, 19, 22, 25, 26], potentially leading to cardiomyopathy over time. Disturbances in intestinal permeability among individuals with CD may facilitate the absorption of luminal antigens or infectious pathogens, ultimately causing damage to the heart through immune-mediated processes. Additionally, an immune response directed at an antigen present in both the heart and the small intestine may result in direct cardiac injury [12, 26–28]. Autoimmune diseases often exhibit a hereditary predisposition, with CD being prevalent in approximately 20% of adults [12].

Several patients who have secondary carnitine deficiency due to CD-related malabsorption even without systemic symptoms of carnitine deficiency were found to have DCM; however, it remains still inconclusive whether carnitine deficiency causes cardiomyopathy or merely an incidental finding seen in chronic malabsorption [21].

In comparison with the controls, individuals with CD exhibited higher systolic blood pressures and lower diastolic blood pressures. This phenomenon may be attributed to a hyperdynamic circulation resulting from iron deficiency anemia in patients experiencing chronic malabsorption [22].

### Cardiac manifestations in celiac disease

In this systematic review, we have analyzed and synthesized the available literature to explore the association between Celiac disease and cardiac manifestations, including myocardial infarction, atrial fibrillation, cardiomyopathy, pericardial effusion [27, 29], and other related conditions.

One significant finding is the heightened risk of myocardial infarction in CD patients. A retrospective cross-sectional study conducted in Sweden, which compared 1075 individuals with biopsy-confirmed CD to a matched sample from the general population, identified a higher odds ratio (OR) of myocardial infarction in CD patients compared to controls [17]. Similarly, Conroy et al. reported an increased hazard ratio for ischemic heart disease and myocardial infarction in individuals with CD [30].

Another notable cardiac condition associated with CD is atrial fibrillation (AF). Research by West et al. demonstrated a slightly elevated risk of AF in CD patients, although not statistically significant [31]. In contrast, a larger study of 28,637 CD patients showed a significantly increased risk of AF [16]. These findings collectively suggest a potential link between CD and AF, though the precise mechanisms remain unclear.

Frustaci and colleagues conducted a case–control study, revealing a significantly higher prevalence of biopsy-confirmed CD in 187 patients and suggested “4.4% incidence of CD in patients with lymphocytic myocarditis” [32]. Conversely, Elfstrom and their team examined the link between CD and myocarditis or pericarditis in a Swedish national registry comprising 9,363 children and 4,969 adults with CD [33]. They found no significant association between CD and myocarditis in childhood (HR 0.2, 95% CI 0.0–1.5) or adulthood (HR 2.1, 95% CI 0.4–11.7). They also did not find a substantial connection between CD and pericarditis in childhood (HR 0.4, 95% CI 0.1–1.9) or adulthood (HR 1.5, 95% CI 0.5–4.0). These differing outcomes may be due to the rarity of myopericarditis in both study populations, underscoring the necessity for further investigation on this topic.

Furthermore, within the spectrum of CD-related myocarditis, Myrmel et al. highlighted the presence of myocarditis as an extra-intestinal manifestation of CD, a finding consistent with some prior studies demonstrating a link between CD and myocarditis. “Prevalence rates in these studies ranged from 1.8 to 5.7%.” Reliable evidence from case reports increases the strength of the association [23, 29, 33–35].

### Association of celiac disease and dilated cardiomyopathy

The association of cardiomyopathy in individuals with CD remains a topic of considerable uncertainty, requiring a comprehensive examination of the existing evidence. Wang et al. contributed valuable insights to our understanding of this association. Curione’s cohort study, which encompassed 52 patients diagnosed with idiopathic DCM, revealed that 5.8% of these patients had biopsy-confirmed CD [17]. These findings mark the consideration of CD as a potential comorbidity in patients with DCM. Noori et al.'s study results support Curione's study by suggesting that children with DCM may have a heightened likelihood of also having CD. Notably, their study detected tissue transglutaminase IgA -positive values in approximately 18% of the patients with DCM, reinforcing the potential connection between these two conditions [15, 21].

However, results from Elfstrom’s Swedish national registry did not find a statistically significant association between cardiomyopathy or CD [33]. Similar results from Emilsson, who conducted a comprehensive investigation involving 29,000 CD patients and 144,429 healthy controls, matched regarding age, sex, and place of residence to evaluate the risk of idiopathic DCM. Interestingly, their results did not show a statistically significant increase in the risk of DCM in patients with CD compared to that of control group. This finding underscores the variability in results across different studies and the need for cautious interpretation. These results seem to contrast with those of Curione et al. [15, 36].

Prati's study added another layer of insight, reporting that 1.9% of patients who were candidates for heart transplantation had positive tissue transglutaminase IgA, whereas only 0.37% in the control group tested positive. This aligns with similar results obtained by Curione, which resonate with the findings of our review (18.42% in the patients and 5.26% in the controls). These variations in seropositivity rates highlight the complexity of CD seropositivity as a potential marker in the context of DCM and emphasize the need for further investigation [15, 21, 37].

Additionally, Rashidinia's research uncovered a higher prevalence of celiac disease seropositivity among DCM patients when compared to the reported prevalence in the general population of Iran. This regional variation in celiac disease seropositivity further underscores the multifaceted nature of this relationship and the potential influence of geographical factors on its manifestation [24].

Furthermore, it is essential to acknowledge the collective findings of Elnour, Bohra, Mehra, Saada, and McGrath which suggest an increased risk of DCM associated with CD. These studies contribute to the growing body of evidence regarding the potential connection between CD and DCM [12, 25, 26, 29, 34].

However, all the varied results highlight the importance of considering the characteristics of the study population when interpreting results. The apparent discrepancy in the findings of these studies prompts us to consider several factors. First, the differing methodologies and study populations may contribute to these discrepancies. Curione et al.'s study focused on a specific cohort of patients with idiopathic DCM, while the Elfstrom’s Swedish national registry study included a broader population of individuals with CD. This highlights the importance of considering the characteristics of the study population when interpreting results.

Second, it is worth noting that the prevalence of CD varies across populations and regions. This variability might influence the observed associations. Moreover, the infrequency of DCM itself may affect the identification of connection. Moreover, the diagnosis of cardiomyopathy and CD may vary in accuracy and criteria between studies ranging from serology alone to biopsy findings, potentially affecting the observed associations.

### Management of cardiac manifestations in celiac disease

Managing cardiac manifestations in celiac disease requires a comprehensive approach. While strict adherence to a gluten-free diet remains fundamental to mitigating associated risks, it is vital to acknowledge the complexity of this relationship as demonstrated by Schmucker et al. (2020), who reported a slight increase in the risk of developing type 2 diabetes with higher gluten consumption, a significant risk factor for cardiac morbidity [38]. However, the overall association between gluten intake and health outcomes remains unclear, based on a review of available evidence with low certainty. This evidence does not strongly support a clear link between gluten consumption and type 2 diabetes risk or cardiac events, or mortality. An included randomized controlled trial found no significant impact of gluten intake on cardiovascular risk factors, including blood pressure, LDL cholesterol, or BMI, although the certainty of these findings is also low. Unfortunately, these studies did not report data on adverse events or other outcomes.

Lebwohl et al. demonstrated that strict adherence to a gluten-free diet is associated with the reversal of histological features of CD and a decrease in immune activation linked to cardiac risk [18]. It has also been shown that a gluten-free diet can lead to the resolution of pleural and pericardial effusions. Additionally, some cases have exhibited improved cardiac function through a gluten-free diet, notably in the reduction in ventricular arrhythmias and improvements in ventricular volumes and ejection fraction [21, 22, 25, 26, 29].

Conversely, Rashidinia et al. found no improvement in cardiac features with a gluten-free diet (GFD) and hypothesized that seropositivity might be influenced by other unrecognized factors shared between CD and cardiomyopathy [24]. However, research on the impact of a gluten-free diet on cardiac health remains ongoing, with variable outcomes reported.

A crucial aspect of managing CD involves addressing chronic inflammation, which appears to correlate with the severity of ventricular arrhythmias in affected patients [4]. Nutritional therapy, including a gluten-free diet and supplementation with nutrients such as iron, calcium, and vitamins, remains the cornerstone of CD management [27, 35]. However, data on cardiac outcomes in CD patients are diverse and sometimes inconclusive demanding further evidence [34].

Hence, a stringent gluten-free diet results in complete or partial recovery in many cases and complete refractoriness in some. In essence, the management of cardiac manifestations in CD necessitates a comprehensive approach that combines dietary interventions, addressing inflammation, and managing associated comorbidities to optimize cardiac health in affected individuals.

### Limitations

It is important to acknowledge certain limitations in this review. The scope of this study was confined to free full-text articles written in the last 10 years, from 2013 to 2023 and written in the English language. This selection protocol aimed to collect comprehensive and up-to-date reviews of literature. However, it may have inadvertently excluded valuable insights from older, non-English, or paid publications. Though efforts were made to ensure the accuracy of the data presented, there may be potential bias in data from the sources selected in the study. Despite these limitations, this review strives to provide valuable insights into the topic of cardiac manifestations in CD, an association of CD with DCM within the limited constraints.

## Conclusions

This systematic review draws the association between celiac disease (CD) and various cardiac manifestations, including dilated cardiomyopathy, myocardial infarction, atrial fibrillation, pericardial effusion, myocarditis, pericarditis, and related conditions. Studies indicate a heightened risk of certain cardiac diseases in individuals with CD, particularly a potential link with dilated cardiomyopathy (DCM). Management involves a multidisciplinary approach with strict adherence to a gluten-free diet, alongside nutritional therapy and inflammation management. Due to the diverse results of this review, it potentiates the need for further research with standardized methodologies, larger sample sizes, and consideration of regional variations to gain a more comprehensive understanding of the complex relationship between CD and cardiac manifestations. Such insights are very crucial for improving clinical practice and ensuring the optimal management of cardiac health in individuals with celiac.

### Supplementary Information

Supplementary information 1

## Data Availability

All data generated or analyzed during this study are included in this published article [and its supplementary information files].

## Abbreviations

CD: Celiac disease
DCM: Dilated cardiomyopathy
