# Supplementary material for: The heart of celiac disease: understanding dilated cardiomyopathy, pathophysiology, and care—a systematic review
Source: Egypt Heart J. 2024 Aug 16;76:107. doi: 10.1186/s43044-024-00534-x (PMC11329460; doi:10.1186/s43044-024-00534-x)
Supplement: Supplementary file 1 — Supplementary information 1 [file 43044_2024_534_MOESM1_ESM.docx]

**SUPPLEMENTARY DATA:**

| TABLE3: Quality assessment for Systematic Reviews |  |  |
| --- | --- | --- |
| AMSTAR checklist | **Hidalgo et al. (2020)** | **Schmucker et al. (2022)** |
| 1. Was an 'a priori' design provided? | yes | yes |
| 2. Was there duplicate study selection and data extraction? | no | no |
| 3. Was a comprehensive literature search performed? | yes | yes |
| 4. Was the status of publication (i.e. grey literature) used as an inclusion criterion? | no | no |
| 5. Was a list of studies (included and excluded) provided? | yes | yes |
| 6. Were the characteristics of the included studies provided? | yes | yes |
| 7. Was the scientific quality of the included studies assessed and documented? | yes | yes |
| 8. Was the scientific quality of the included studies used appropriately in formulating conclusions? | yes | yes |
| 9. Were the methods used to combine the findings of studies appropriate? | yes | yes |
| 10. Was the likelihood of publication bias assessed? | yes | yes |
| 11. Was the conflict of interest included? | yes | yes |
| Score (included) | 9(yes) | 9(yes) |

| Table4 : Quality assessment for Narrative review |  |
| --- | --- |
| SANRA checklist | Wang et al. (2023) |
| 1) Justification of the article’s importance for the readership | yes |
| 2) Statement of concrete aims or formulation of questions | yes |
| 3) Description of the literature search | yes |
| 4) Referencing | yes |
| 5) Scientific reasoning | yes |
| 6) Appropriate presentation of data | yes |
| score | 6 |

| Table5: NEWCASTLE - OTTAWA QUALITY ASSESSMENT SCALE - CASE CONTROL STUDIES | | |
| --- | --- | --- |
|  | Noori et al. (2016) | Huang et al. (2022) |
| SELECTION |  |  |
| 1) Is the case definition adequate? | * | * |
| 2) Representativeness of the cases | * | * |
| 3) Selection of Controls | - | * |
| 4) Definition of Controls | * | * |
| compatibility |  |  |
| 1) Comparability of cases and controls on the basis of the design or analysis | * | * |
| EXPOSURE |  |  |
| 1) Ascertainment of exposure | * | * |
| 2) Same method of ascertainment for cases and controls | * | * |
| 3) Nonresponse rate | - | * |
| score | 6 | 8 |
| accept | yes | Yes |

| Table6 : NEWCASTLE - OTTAWA QUALITY ASSESSMENT SCALE – CROSS SECTIONAL STUDIES | | | | |
| --- | --- | --- | --- | --- |
|  | Rashidinia et al. (2021) | Karadas et al. (2016) | Ibrahim et al. (2023) | Lebwohl et al. (2015) |
| SELECTION |  |  |  |  |
| 1) Is the case definition adequate? | * | * | * | * |
| 2) Representativeness of the cases | * | * | * | * |
| 3) Selection of Controls | * | * | * | * |
| 4) Definition of Controls | * | * | * | * |
| compatibility |  |  |  |  |
| 1) Comparability of cases and controls on the basis of the design or analysis | * | * | * | * |
| EXPOSURE |  |  |  |  |
| 1) Ascertainment of exposure | * | * | * | * |
| 2) Same method of ascertainment for cases and controls | * | * | * | * |
| 3) Nonresponse rate | * | * | * | * |
| score | 8 | 8 | 8 | 8 |
| accept | yes | yes | yes | yes |

|  | Table7: Quality assessment for case reports | | | | | | |  |  |
| --- | --- | --- | --- | --- | --- | --- | --- | --- | --- |
| **JBI Critical Appraisal Checklist** | **1.      Were patient’s demographic characteristics clearly described?** | **2.      Was the patient’s history clearly described and presented as a timeline?** | **3.      Was the current clinical condition of the patient on presentation clearly described?** | **4.      Were diagnostic tests or assessment methods and the results clearly described?** | **5.      Was the intervention(s) or treatment procedure(s) clearly described?** | **6.      Was the post-intervention clinical condition clearly described?** | **7.      Were adverse events (harms) or unanticipated events identified and described?** | **8.      Does the case report provide takeaway lessons?** | **Overall appraisal (Included):** |
| Mehra et al. (2022) | yes | yes | yes | yes | yes | yes | no | yes | yes |
| Bohra et al. (2020) | yes | yes | yes | yes | yes | yes | no | yes | yes |
| Myrmel et al. (2021) | yes | yes | yes | yes | yes | yes | no | yes | yes |
| Samy et al. (2017) | yes | yes | yes | yes | yes | yes | no | yes | yes |
| Ashrafi et al. (2014) | yes | yes | yes | yes | yes | yes | no | yes | yes |
| Saada et al. (2017) | yes | yes | yes | yes | yes | yes | no | yes | yes |
| Elnour et al. (2017) | yes | yes | yes | yes | yes | yes | no | yes | yes |
| Patel et al. (2018) | yes | yes | yes | yes | yes | yes | no | yes | yes |
